# Engaging a polylactide copolymer in oral tissue regeneration: first validation of Suprathel^®^ for guided epithelial and osseous healing

**DOI:** 10.25122/jml-2021-0083

**Published:** 2021

**Authors:** Sergiu Vacaras, Grigore Baciut, Dan Gheban, Simion Bran, Horatiu Colosi, Septimiu Toader, Daiana Opris, Winfried Kretschmer, Avram Manea, Gabriel Armencea, Mihaela Baciut, Horia Opris, Ileana Mitre, Mihaela Hedesiu, Cristian Dinu

**Affiliations:** 1. Department of Maxillofacial Surgery and Radiology, Division of Maxillofacial Surgery and Implantology, Iuliu Hatieganu University of Medicine and Pharmacy, Cluj-Napoca, Romania; 2. Department of Morphological Sciences, Division of Pathoanatomy, Iuliu Hatieganu University of Medicine and Pharmacy, Cluj-Napoca, Romania; 3. Department of Medical Education, Division of Medical Informatics and Biostatistics, Iuliu Hatieganu University of Medicine and Pharmacy, Cluj-Napoca, Romania; 4. Center for Experimental Surgery, Iuliu Hatieganu University of Medicine and Pharmacy, Cluj-Napoca, Romania; 5. Klinik fur Mund-, Kiefer- und Plastische Gesichtschirurgie, Alb Fils Kliniken GmbH, Goppingen, Baden-Wurttemberg, Germany; 6. Department of Maxillofacial Surgery and Radiology, Division of Maxillofacial Radiology, Iuliu Hatieganu University of Medicine and Pharmacy, Cluj-Napoca, Romania

**Keywords:** Suprathel^®^, guided tissue regeneration, oral mucosa, socket preservation, bone healing, CPB – porcine bone, FDBA – frozen-dried bone allograft, GBR –Guided bone regeneration, PCL – polycaprolactone, PGA – poly-glycolic acid, PLA – poly-lactic acid, PLGA – poly-lactide-co-glycolide, PVA – polyvinyl alcohol, rhBMP-2 – recombinant human morphogenetic protein-2

## Abstract

The present study investigated the capacity of Suprathel^®^ (a copolymer membrane, so far validated for skin regeneration) to also regenerate oral tissue – mucosa and bone, by comparing this biomaterial, in a split-mouth rabbit model, to Mucoderm^®^, a xenogeneic collagen matrix certified for keratinized oral mucosa healing. The clinical reason behind this experimental animal model was to determine whether the benefits of this advanced skin regeneration product (Suprathel^®^) could be conveyed for future evaluation in clinical trials of oral tissue regeneration in humans. The outcomes of this study validated the use of Suprathel^®^, a terpolymer of polylactide with trimethylene carbonate and ε-caprolactone, for stimulation of oral epithelium and alveolar bone regeneration in rabbits. Both Suprathel^®^ and Mucoderm^®^ exhibited comparable results and the null hypothesis stating a comparable regenerating effect of these two materials could not be rejected.

## Introduction

Oral tissue regeneration is a sophisticated blend of intricated processes occurring continuously due to age, pathologic deterioration of gingiva and bone, trauma, and teeth loss. Considering the functional and anatomic significance of the involved regions, the deficiency can range from minor to significant or even life-altering.

Teeth loss is a process that determines resorption of the alveolar bone, which is clinically and radiologically evident by the loss of bone height and width [1]. The current state of the art of treating edentulous ridges is by using implant-supported prostheses [2]. The prosthetically-driven planning involves the correct three-dimensional (3D) positioning of dental implants, representing the key to therapeutic success [3].

Bone resorption occurs in time, but most of the bone is lost during the first 4 weeks after the tooth is lost, averaging 3–5 mm in width after 6 months [4]. Bone regeneration and other surgical augmentation procedures have been used with a high degree of success to restore the alveolar process and prepare the tissues for dental implant therapy with correct 3D positioning [5]. Consequently, after tooth extraction, it is highly beneficial to preserve the remaining bone and stop the bone resorption process [6]. Socket preservation procedures have long been studied and implemented, but a consensus regarding the best method of preserving the bundle bone, using various types of bone substitute material, with or without barrier membranes, is still lacking [5, 7].

### Tissue regeneration with an ideal barrier membrane

A guided bone regeneration membrane needs to create and maintain a secluded space, protecting an environment favorable to the bone-forming cells, sheltered from the epithelial cells [8]. The ideal characteristics of the barrier membranes used in such cases need to be biocompatible, provide cell occlusion, integration by the host, clinical manageability, and space-making abilities [9]. Although several advantages of using resorbable membranes exist, such as no need for a second surgery, improved cost-effectiveness, and simplified surgical protocol, non-resorbable membranes still serve as the gold standard of bone regeneration [10].

### Gingival recession/lack of keratinized tissue

Morphological characteristics of the oral mucosa are considered an important factor in the stability and integrity of the periodontium [11]. The non-keratinized mucosa facilitates the penetration of bacteria into the crevice, causing an inflammatory response with subsequent attachment loss [12]. It has long been suggested that an adequate zone of keratinized tissue was critical for preserving the stability of periodontal tissues [13]. Recent studies have suggested that a wider band of keratinized gingiva may determine significant differences concerning the lowering of the plaque index, the values of the modified gingival index, the mucosal recession, and attachment loss [14]. Lack of attached keratinized mucosa was linked to hygiene discomfort and lower vestibular depth [15, 16].

### Purpose of the study ^–^ Suprathel®

The purpose of the current investigation was to assess the effectiveness of a polylactide copolymer (Suprathel^®^) in oral tissue regeneration and stimulate innovative research to promote oral tissue regeneration. Before this study, the split-mouth design used to compare the proposed material to a long-time validated product (Mucoderm^®^) has been perfected using a preliminary pilot study on only two rabbits, that has been implemented by the same research team [17], in order to optimize the assessment criteria of tissue regeneration, to yield reproducible outcome data and to allow an objective statistical appraisal of the experimental results. Perfecting this split-mouth design and its assessment criteria was also aimed at a possible translation of this preclinical experience to future clinical research.

### Connective tissue graft Mucoderm®

The use of a connective tissue graft in enhancing the width of the keratinized tissue is considered the standard of care [18]. Also, the use of the graft enhances the marginal tissue thickness to improve the long-term stability of the periodontium [19]. The downsides are that it complicates the surgical procedure, increases morbidity, and raises the rates of wound failure during early healing. To try to balance off the latter, alternatives have been studied, such as human dermis and collagen matrices [20]. Xenogeneic matrices, like Mucoderm^®^, have several advantages, such as no need for human donors, no risks of transmitting diseases, and no limit to the size of the surgical site size [21]. Recent developments have indicated that xenogeneic collagen matrices can provide soft tissue enhancement similar to connective tissue grafts and also have an improved patient-reported outcome [22].

In search for consolidating the clinical applicability of a proposed approach, the role and benefit of the method and its effect have to be thoroughly inspected, starting on animal models.

## Material and Methods

### Study design

Twenty New Zealand rabbits (n=20) from the Biobase of the Iuliu Hațieganu University of Medicine and Pharmacy in Cluj-Napoca, Romania, were used in this study. All rabbits were 2 years-old males and weighed between 3–4 kilograms.

The sample size was chosen in line with accepted guidelines for sample size calculation in animal studies, according to which the degree of freedom of an analysis of variance, E, should lie between 10 and 20, where E = total number of animals – total number of groups [23]. All animals that showed signs of infection or died during the course of the experiment were excluded.

### Randomization and blinding

The study aimed to evaluate both epithelial and bone regeneration. For this reason, it was structured based on two experimental arms that were created and followed in all 20 rabbits, using a split-mouth pattern according to a blinding protocol designed to compare epithelial and bone regeneration of the two investigated materials.

**Figure 1.**
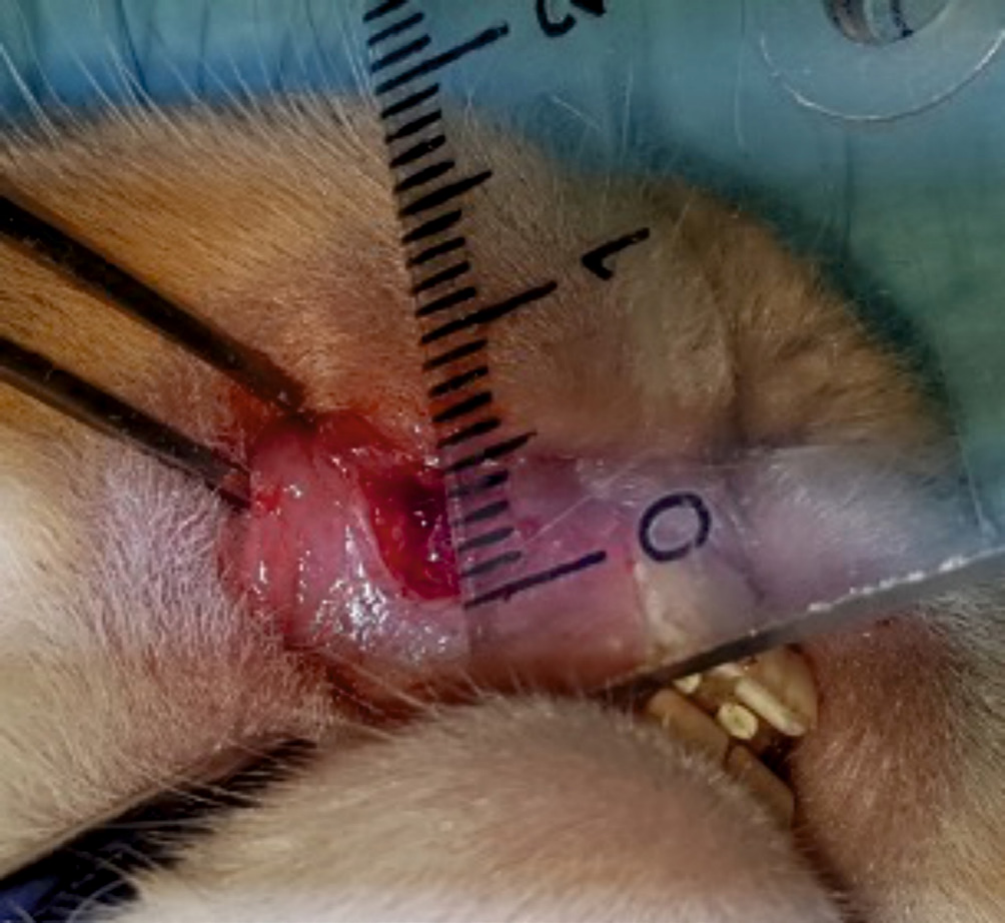
Creation of 5x5 mm mucosal defects in the upper anterior vestibule (bilaterally).

The arm of the study investigating the epithelial regeneration potential of the two materials involved the creation of 5x5 mm mucosal defects in the upper anterior vestibule bilaterally in the oral cavity of all 20 rabbits (Figure 1).

**Figure 2.**
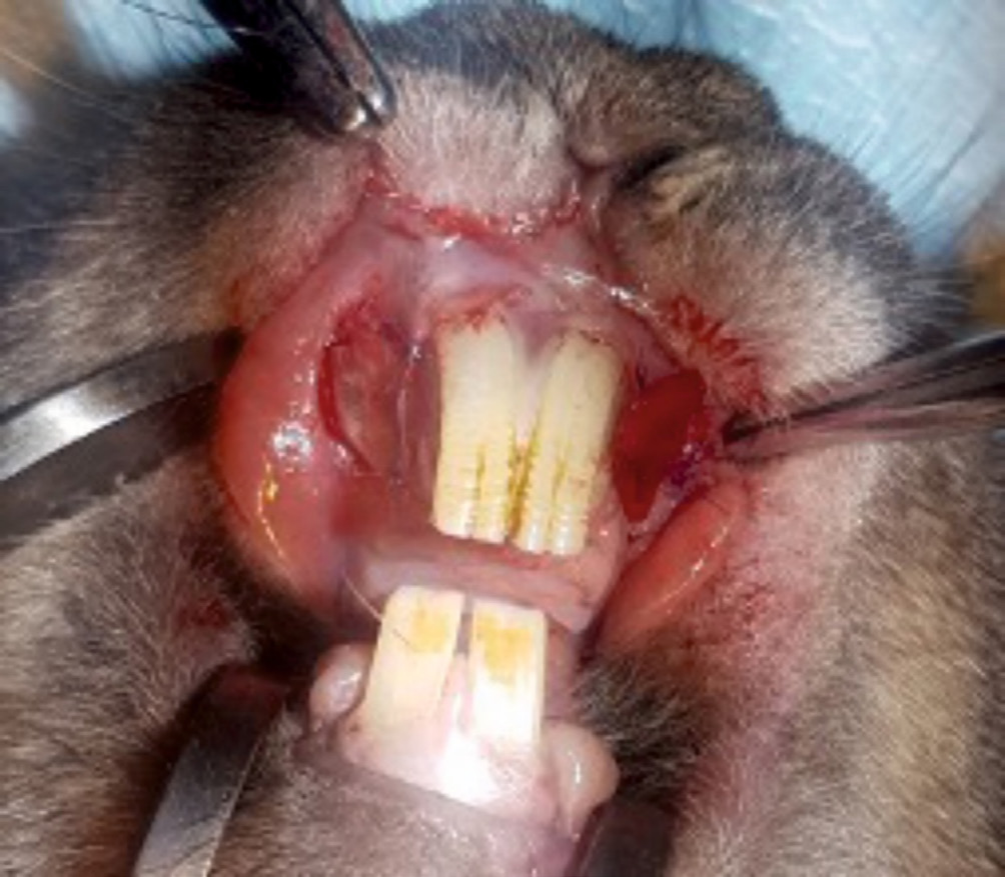
Coverage of mucosal defects with either one of the compared materials on each side.

**Figure 3.**
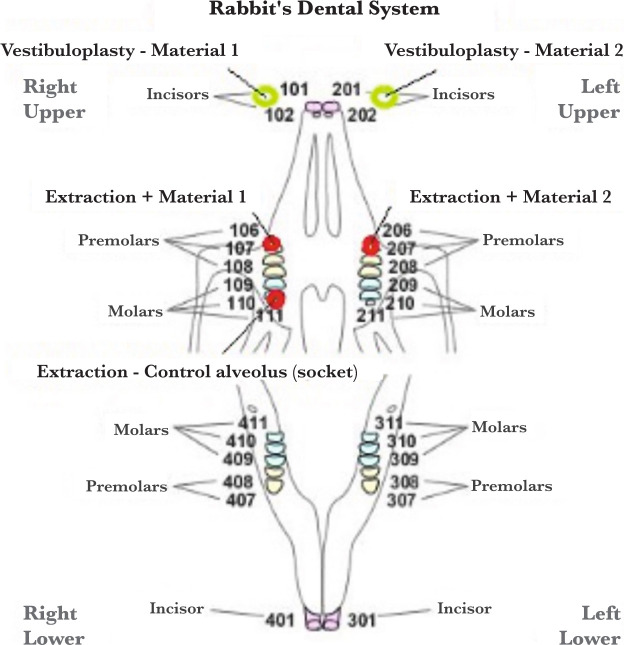
Anatomic scheme of a rabbit’s dental system. The green circles mark the paired sites of vestibuloplasty. The red circles mark the sites of dental extractions.

**Figure 4.**
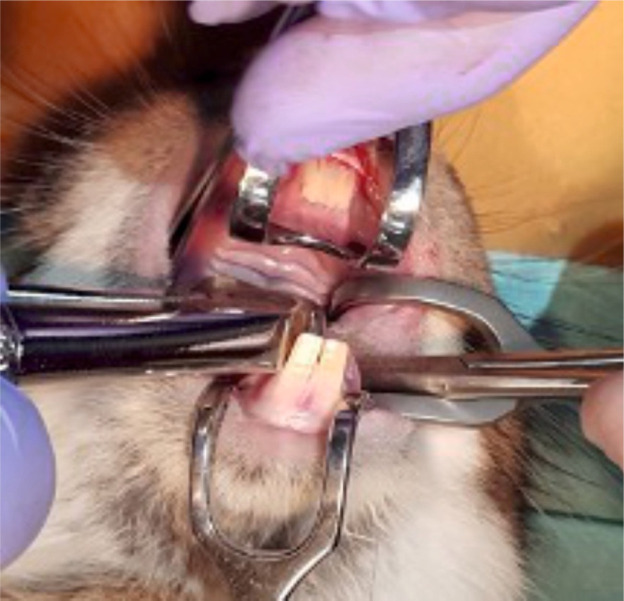
Extraction of maxillary teeth.

**Figure 5.**
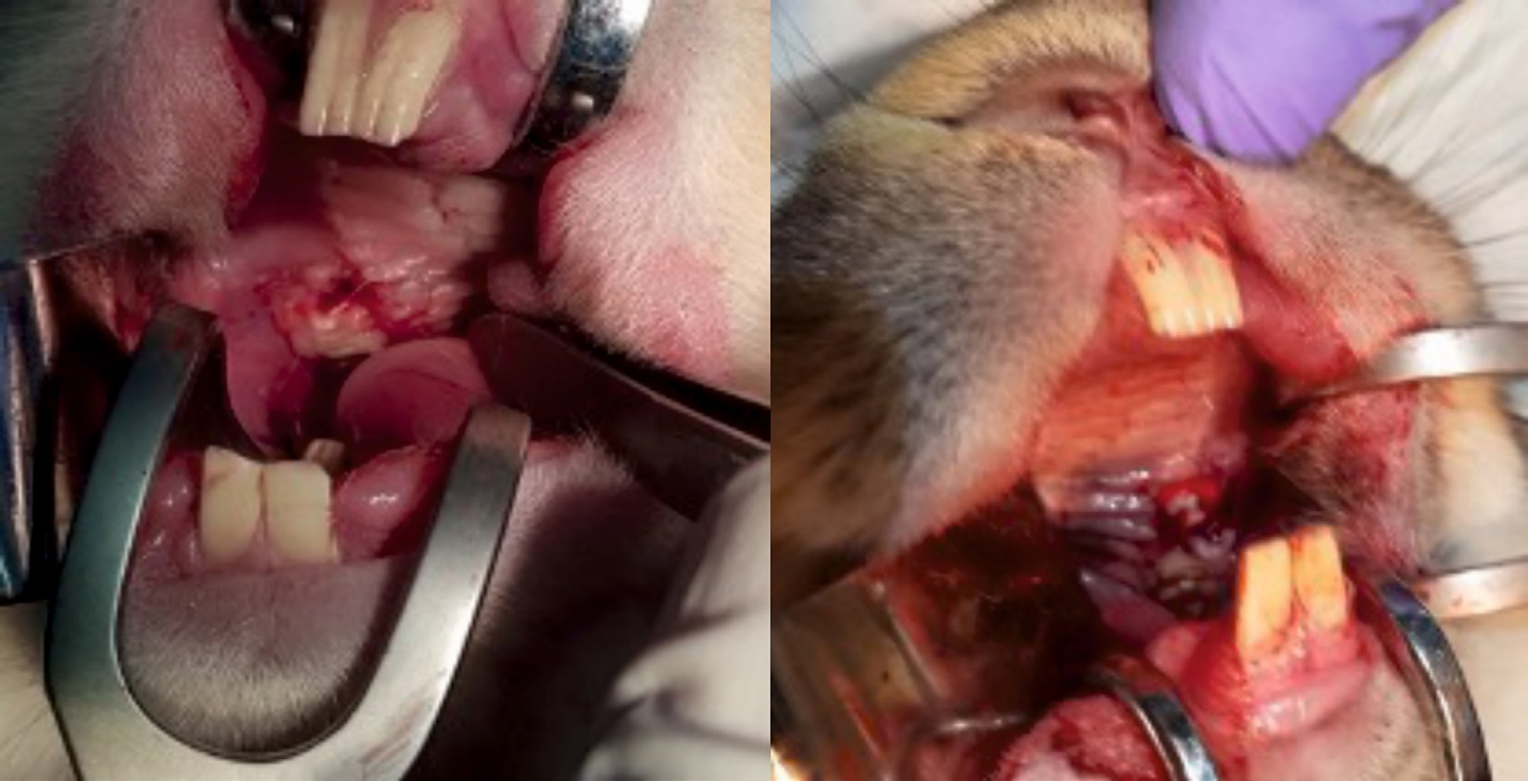
Coverage of the paired alveoli after tooth extractions on each side – the split-mouth pattern.

**Figure 6.**
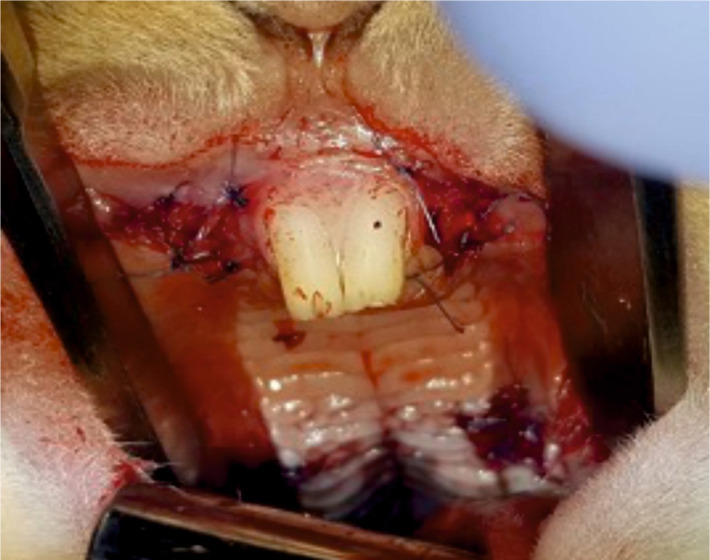
Complete coverage of all reconstruction sites: buccal mucosa and alveolar sockets bilaterally.

The defects were covered using one of the materials on each side (Figure 2). The second arm of the study assessed bone healing. To this end, the interventions involved bilateral extractions of two maxillary premolars on each side, as well as of one molar on one of the sides (Figures 3, 4), followed by alveolar coverage with the investigated materials, except for the molar socket, which was left uncovered, as a control socket with spontaneous healing (Figures 5 and 6).

All surgical interventions took place under general anesthesia, which was performed using 10% ketamine (Rotexmedica GmbH Arzneimittelwerk, Trittau, Germany) and 2% xylazine (Bioveta, Ivanovice na Hane, Czech Republic) in a ratio of 2:1. All the involved procedures were in accordance with the Animal Research: Reporting of In Vivo Experiments (ARRIVE) guidelines and the ethical standards comprised in the Institutional and National Guide for the care and use of laboratory animals.

The two materials used for coverage were: a specially fabricated, double thickness (250 microns thick) Suprathel^®^ membrane patch (Polymedics Innovations, Denkendorf, Germany) and a commercially available Mucoderm^®^ membrane patch (Botiss Biomaterials GmbH, Zossen, Germany).

Neither the investigators who performed the surgical interventions nor those who collected or later analyzed the clinical and histological data knew the assignment of each material for mucosal defect and alveoli coverage, respectively. This way, the blinding protocol was followed in both arms of the study. One of the authors (M.B.) was strictly in charge of controlling the preservation of blinding and the use of each material for every operated site.

The harvesting of specimens was performed three months later, after euthanasia of the animal using Vetased (SC Pasteur Filiala Filipești, Filipeştii de Pădure, Romania) in overdose.

**Figure 7.**
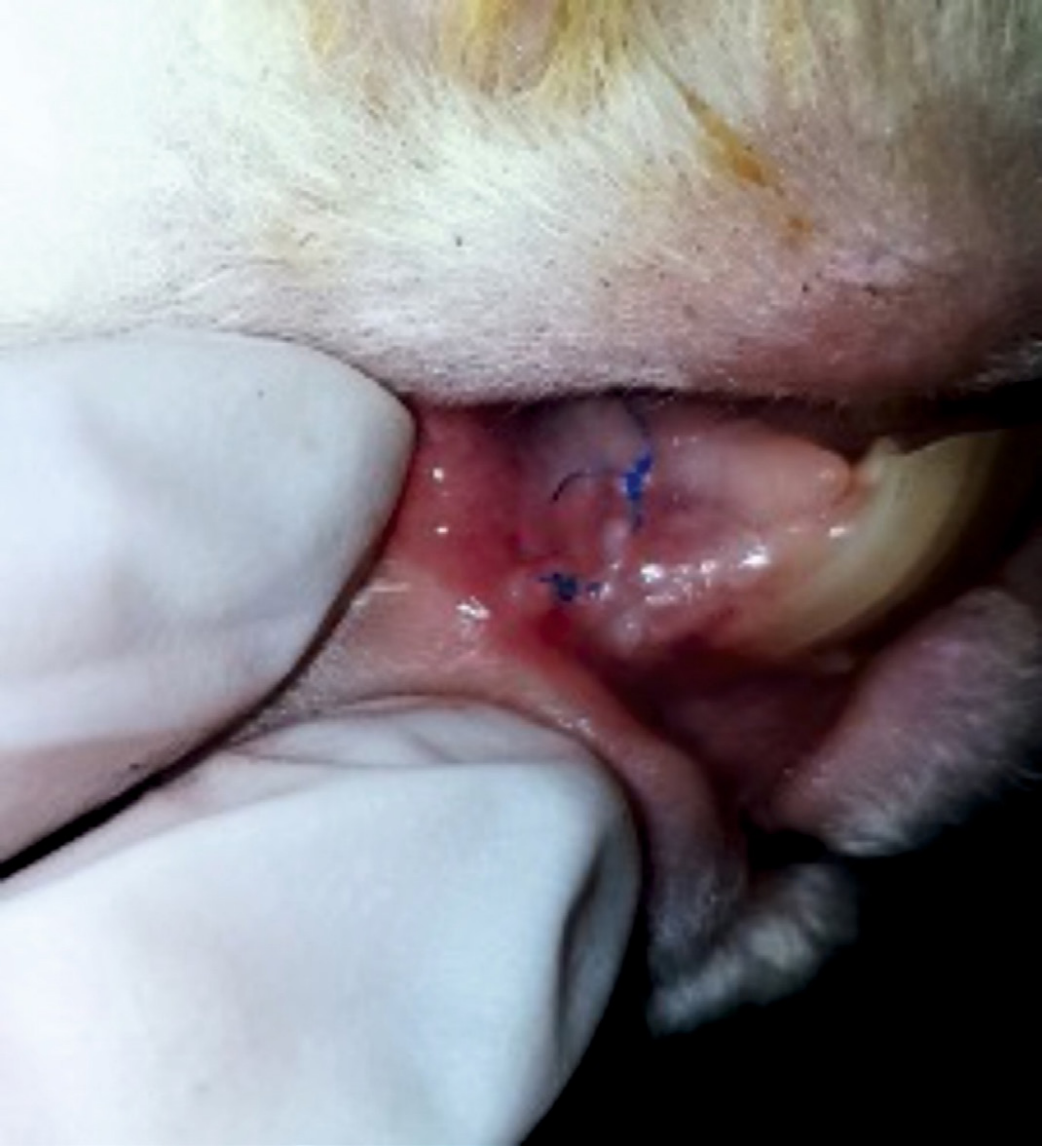
Vestibulum site at 3 months postoperatively with clinically healed mucosa, ready for the evaluation of the guided tissue regeneration (GTR).

**Figure 8 (A, B, C).**
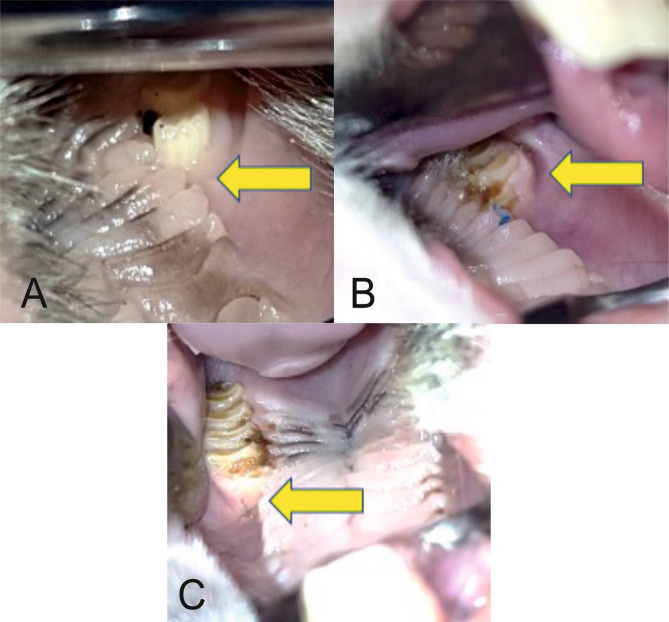
Healed postextraction alveoli (arrows).

The maxillae with the mucosal and alveolar regeneration sites were evaluated clinically for the absence of pathological signs and were resected, after euthanasia, in order to perform a histological evaluation of the mucosal healing process and the quality and quantity of alveolar bone healing (Figures 7 and 8 a, b, c).

Histological staining was performed for all operation sites with hematoxylin-eosin and according to Masson’s trichrome technique. All sections were examined by a histopathologist with 25 years of experience in assessing guided oral tissue regeneration (G.D.) and by three other investigators (A.M; C.D.; H.O.). The histological regeneration of the epithelial defects and bone healing were independently assessed by each of these four investigators.

The evaluation process was planned based on criteria established in a previous pilot study, from which the array of the most useful aspects for characterization of the newly formed tissue emerged [17]. Comparison with spontaneous healing of the mucosal defect has not been performed because of the cicatricial spontaneous healing with retractile connective scarring of the tissue, without any possibility for a meaningful comparative evaluation to guided tissue regeneration (GTR).

Data for histologic evaluation of the epithelial healing comprised the following parameters: the maximum and minimum thickness of the superficial layer (in micrometer), the total number of pathologic foci, presence of normal, hyper- and hypoplastic (with reduced spinous layer) epithelium, presence of inflammation, simple granuloma, as well as foreign body granuloma in distinct microscopic fields.

As for the bone healing evaluation criteria, the following aspects were recorded and analyzed after being quantified on a 5-points Likert scale: normal bone trabeculae, bone formation (filling of alveolus with bone), vascularization (neoformation vessels), mature bone, structural integrity at the membrane surface, presence of inflammation/granulation tissue, material persistence, presence of abscesses in the post-extraction site, newly formed cartilaginous tissue, newly formed fibrous tissue.

The 5-points Likert scale enabled the estimation of 5 categories, ranging from the absence of the criterion to the extremely intense/clear/generalized presence of the criterion.

### Data analysis

Descriptive statistics have been computed and reported as the median and interquartile range (IQR), given the skewed distribution of the studied dataset. Mean and standard deviation have also been reported for contrasting and an improved overview of these descriptive results.

Inferential statistics has investigated three main categories of questions, pertaining to the epithelial (mucosal) healing study arm and the bone healing study arm.

Firstly, the current study tested the null hypotheses that no differences appeared during the mucosal healing process between the two groups (Mucoderm^®^ vs. Suprathel^®^) concerning the frequency of the following histological characteristics:

• Presence of superficial epithelialization;
• Presence of profound epithelialization;
• Presence of remnant membrane material.

The above hypotheses have been tested by applying McNemar’s test on the corresponding contingency tables of these paired observations.

Secondly, the study tested the null hypotheses that no differences appeared after the mucosal healing process between the two groups (Mucoderm^®^ vs. Suprathel^®^) concerning the minimum and the maximum thickness of the superficial epithelial layers of the healed regions, as well as the total number of identifiable pathological foci.

Since these measurements did not follow normal distributions, Wilcoxon tests for paired datasets have been used to test these hypotheses.

Thirdly, the null hypotheses were tested that no differences appeared during the healing process concerning the Likert scoring of the following histological characteristics in the compared groups (Mucoderm^®^ vs. Suprathel^®^):

• Normal epithelium;
• Hyperplasic epithelium;
• Hypoplasic epithelium;
• Inflammation;
• Simple granuloma;
• Foreign body granuloma.

Once again, Wilcoxon tests for paired datasets have been used to test these hypotheses because the recorded Likert scores were not normally distributed. Finally, the healing after extraction has been compared between the three alveoli (two alveoli covered with Mucoderm^®^ and Suprathel^®^ and a third one left for natural healing), concerning the following characteristics, quantified by Likert scores:

• Presence of normal bone trabeculae;
• Bone-filled alveoli;
• Bone maturity;
• Vascularization/vascular neo-formation;
• Presence of inflammatory tissue;
• Presence of abscesses in the post-extraction site;
• Newly formed cartilaginous tissue;
• Newly formed fibrous tissue;
• Structural integrity at the contact point with the membrane (only for the alveoli covered with Mucoderm^®^ and Suprathel^®^);
• Persistence of membrane material (only for alveoli covered with Mucoderm^®^ and Suprathel^®^).

Freedman tests and post-hoc Wilcoxon tests for paired samples have been used to investigate possible differences in the above-mentioned Likert scores between the investigated alveoli.The threshold for statistical significance has been chosen at a level α=0.05 for all tested hypotheses, with the exception of post-hoc hypotheses, for which a Bonferroni-corrected threshold α=0.0169 was used. Data were collected using Microsoft Excel, and analyses have been performed using IBM Statistical Package for the Social Sciences (SPSS) Statistics v.25 (IBM, Armonk, New York, United States of America). The collected data regarding mucosal epithelial healing and bone regeneration were described and analyzed under blind coding. Then, after unblinding, the obtained results were attributed to each of the studied materials (Suprathel^®^ and Mucoderm^®^).

## Results

The animals had an uneventful recovery after the surgical interventions. One rabbit died on the 9^th^ postoperative day, and no relation could be found with the experiment. An overview of mucosal epithelial healing characteristics under the influence of Mucoderm^®^ and Suprathel^®^ is presented in Table 1.

**Table 1.** Characteristics of epithelial healing guided by the two guided tissue regeneration – GTR materials.

	Mucoderm®				Suprathel®				P-value (Wilcoxon signed ranks test)
	Mean	Std. Deviation	Median	IQR	Mean	Std. Deviation	Median	IQR	
**Max. thickness of the superficial layer (micrometer)**	38.42	17.799	40.00	10.00	32.11	19.099	30.00	20.00	0.168
**Min. thickness of the superficial layer (micrometer)**	16.05	6.364	20.00	10.00	13.16	6.283	15.00	15.00	0.149
**Total number of pathologic foci**	0.53	0.841	0.00	1.00	0.26	0.562	0.00	0.00	0.206
**Hyperplastic epithelium (Likert)**	2.68	1.293	2.00	2.00	2.58	1.427	3.00	2.00	0.711
**Normal epithelium (Likert)**	1.16	1.302	1.00	2.00	1.05	1.353	1.00	2.00	0.873
**Hypoplastic epithelium (Likert)**	0.16	0.375	0.00	0.00	0.37	0.955	0.00	0.00	0.480
**Inflammation (Likert)**	4.00	0.000	4.00	0.00	3.79	0.918	4.00	0.00	0.317
**Simple granuloma (Likert)**	3.89	0.459	4.00	0.00	3.95	0.229	4.00	0.00	0.655
**Foreign body granuloma (Likert)**	3.89	0.459	4.00	0.00	4.00	0.000	4.00	0.00	0.317

### Results of data analysis

The null hypothesis concerning the presence of profound epithelialization has been rejected (p=0.031 – McNemar test). Profound epithelialization was present in a significantly higher number of rabbits on the Mucoderm^®^ side (7 rabbits) compared to the Suprathel^®^ side of the mouth (1 rabbit), as presented in Table 2.

**Table 2.** Contingency table of profound epithelialization depending on the intervention side.

Profound epithelialization Mucoderm®	Profound epithelialization Suprathel®	
	Absent	Present
**Absent**	12	0
**Present**	6	1

None of the other null hypotheses could be rejected, as listed below:

• Presence of remnant membrane material (p=1 – McNemar test);
• Minimum thickness of the superficial epithelial layers (p=0.149 – Wilcoxon test);
• Maximum thickness of the superficial epithelial layers (p=0.168 – Wilcoxon test);
• Total number of identifiable pathological foci (p=0.206 – Wilcoxon test);
• Likert scores regarding:
  ○ Normal epithelium (p=0.873 – Wilcoxon test);
  ○ Hyperplasic epithelium (p=0.711 – Wilcoxon test);
  ○ Hypoplasic epithelium (p=0.480 – Wilcoxon test);
  ○ Inflammation (p=0.317 – Wilcoxon test);
  ○ Simple granuloma (p=0.655 – Wilcoxon test);
  ○ Foreign body granuloma (p=0.317 – Wilcoxon test).

**Figure 9.**
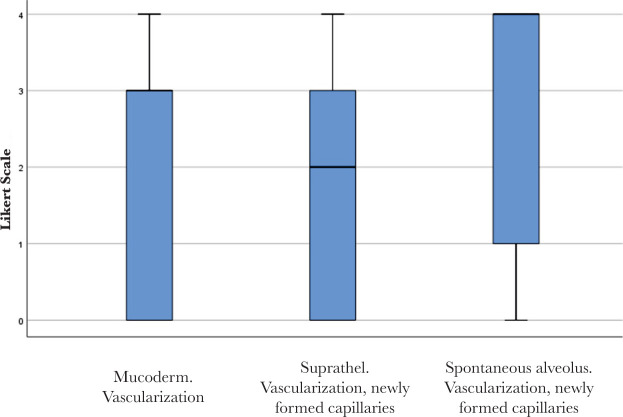
Vascularization and newly formed capillaries in the healing of the three compared alveoli. Bolded lines mark the median Likert score; upper/lower whiskers mark the limits of the largest/smallest values that are not greater/smaller than the third/first quartile plus/minus 1.5 times the interquartile range.

**Figure 10.**
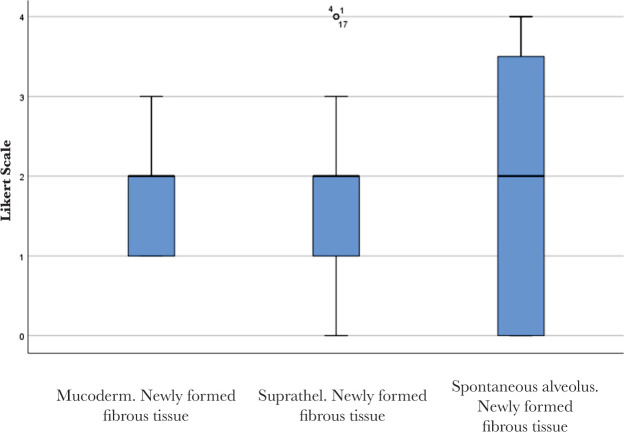
Newly formed fibrous tissue in the healing of the three compared alveoli. Bolded lines mark the median Likert score; upper/lower whiskers mark the limits of the largest/smallest values that are not greater/smaller than the third/first quartile plus/minus 1.5 times the interquartile range.

**Figure 11.**
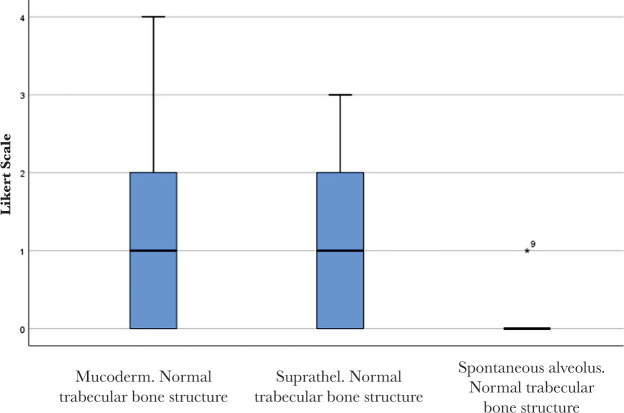
Normal trabecular bone structure in the healing of the three compared alveoli. Bolded lines mark the median Likert score; upper/lower whiskers mark the limits of the largest/smallest values that are not greater/smaller than the third/first quartile plus/minus 1.5 times the interquartile range.

**Figure 12.**
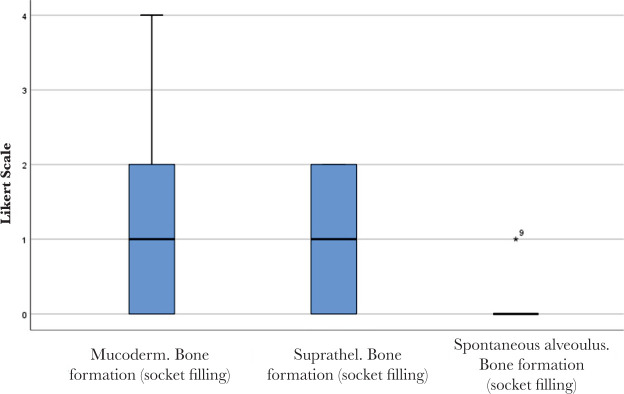
Bone formation (socket filling) in the healing of the three compared alveoli. Bolded lines mark the median Likert score; upper/lower whiskers mark the limits of the largest/smallest values that are not greater/smaller than the third/first quartile plus/minus 1.5 times the interquartile range.

**Figure 13.**
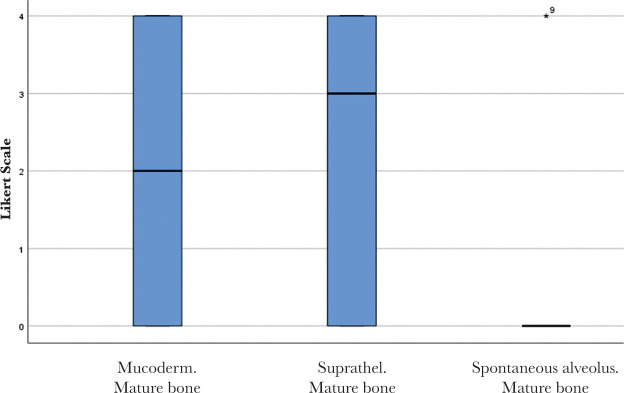
Bone maturity in the healing of the three compared alveoli. Bolded lines mark the median Likert score; upper/lower whiskers mark the limits of the largest/smallest values that are not greater/smaller than the third/first quartile plus/minus 1.5 times the interquartile range.

No significant differences were found between Mucoderm^®^ and Suprathel^®^ concerning any of the alveolar healing characteristics quantified by Likert scores in this study. Figures 9 and 10 illustrate two of these highly comparable characteristics: newly formed capillaries and newly formed fibrous tissue. Nevertheless, both Mucoderm^®^ and Suprathel^®^ exhibited significantly higher Likert scores compared to the naturally healing alveolus in respect to the following three characteristics (Figures 11–13):

○ Presence of normal bone trabeculae (p=0.004/p=0.003 – post hoc Wilcoxon test);
○ Bone-filled alveoli (p=0.004/p=0.003 – post hoc Wilcoxon test);
○ Bone maturity (p=0.006/p=0.002 – post hoc Wilcoxon test).

### Histological aspects of mucosal healing (vestibuloplasty)

**Figure 14.**
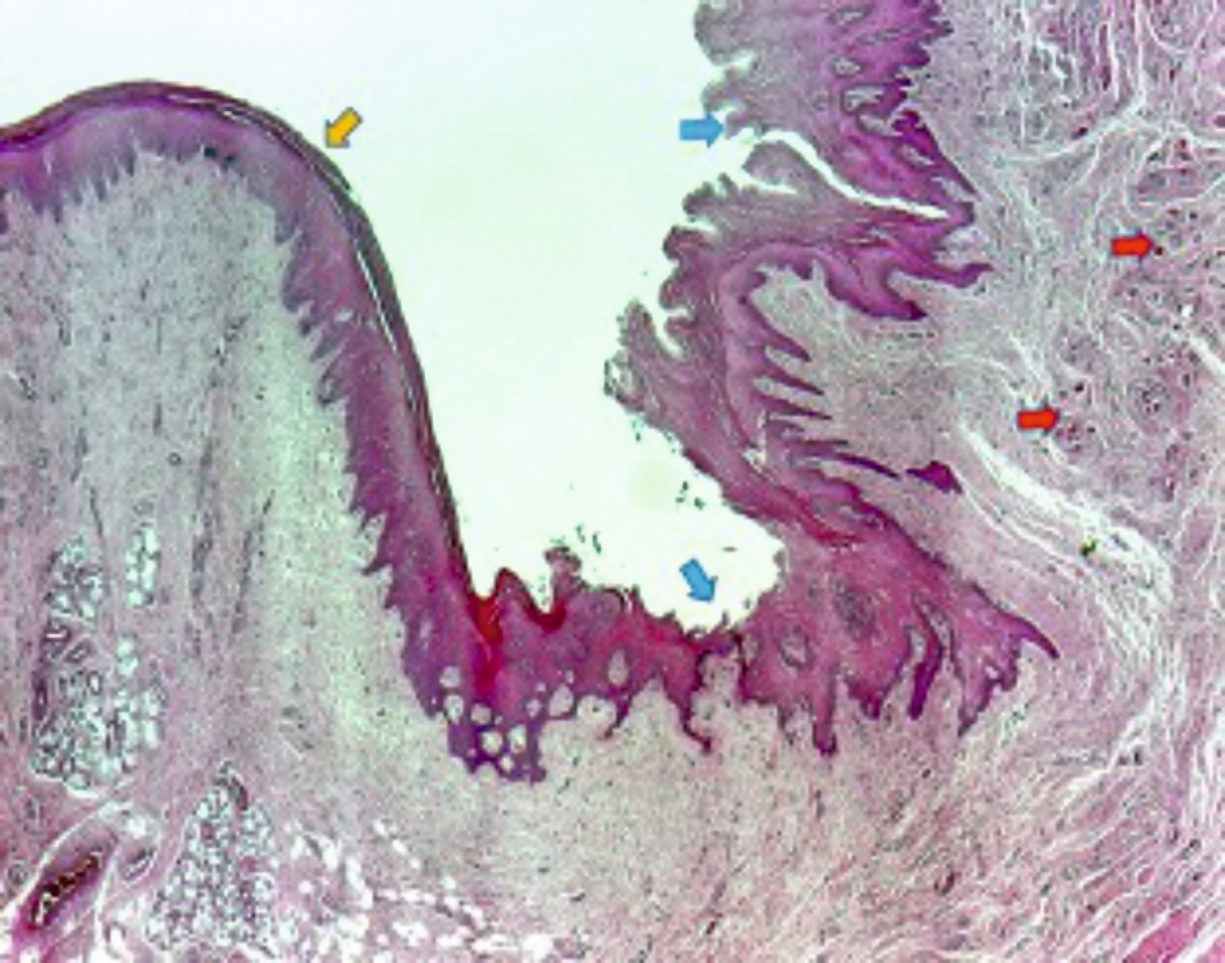
Red arrows indicate towards the rests of Mucoderm® included in the fibrous tissue. Blue arrows point to the new hyperplasic and hyperkeratotic epithelium. The yellow arrow shows the original normal gingival epithelium, with a reduced amount of lamellar keratin at the surface.

**Figure 15.**
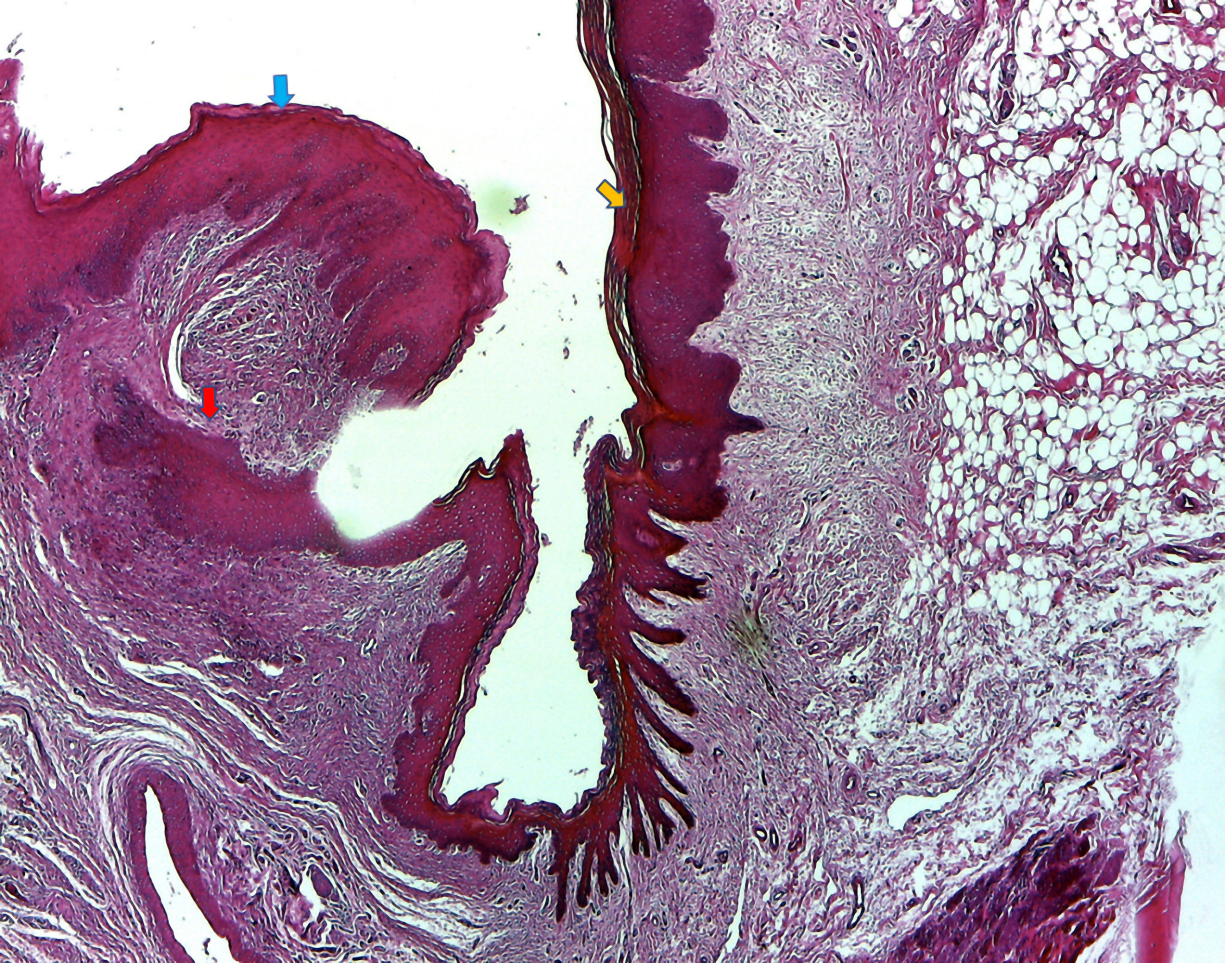
Suprathel® produced the same type of hyperplasic epithelium but less hyperkeratotic (blue arrow) compared to the normal epithelium (yellow arrow). Epithelial extensions surrounded by dense fibrous tissue were seen in the chorion (red arrow).

With regard to the histological results, epithelialization occurred in all cases. Inflammatory hyperplasia with a tendency of hyperkeratosis with orthokeratosis was found more expressed for Mucoderm^®^ and less for Suprathel^®^ (Figures 14, 15). In most cases, Suprathel^®^ was characterized by local epithelial hyperplasia without any other changes (Figure 15).

### Histological aspects of bone healing (extraction socket)

Both materials induced epithelialization on the contour of the dental alveolus and fibrosis (with incorporated particles of the biomaterial in the medullary space of the alveolar bone (in the case of Mucoderm^®^) (Figure 16). Regarding Suprathel^®^, epithelialization occurred in the alveolus with nonkeratinized hyperplasic epithelium, while the biomaterial itself was no longer visible (Figure 17).

**Figure 16.**
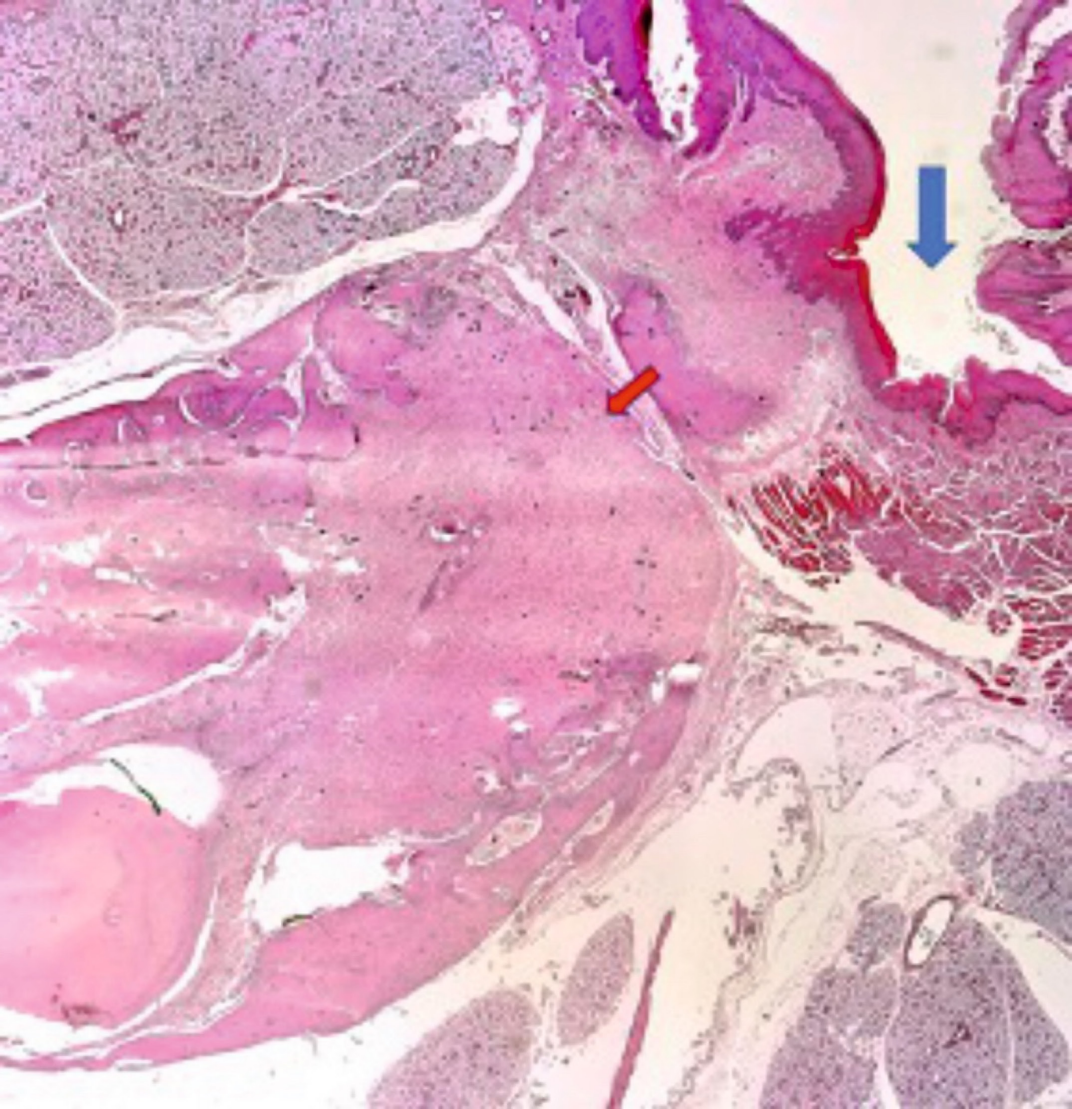
The blue arrow indicates the extraction socket covered with Mucoderm®, epithelialized with keratinized hyperplasic epithelium (similar hyperplasic character as in mucosal healing). The red arrow points to a fibrous nodule containing the included biomaterial formed in the alveolar bone.

**Figure 16.**
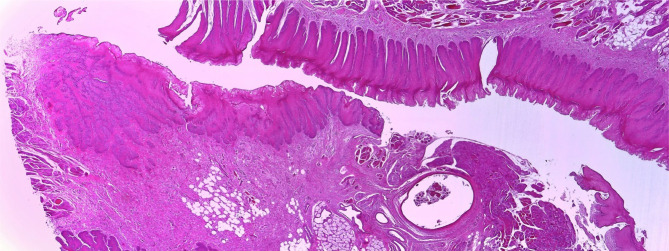
Epithelialized alveolus after coverage with Suprathel®. Nonkeratinized hyperplasic epithelium.

## Discussion

The aim of the present study has been reached by investigating whether the benefits of an advanced skin regeneration biomaterial (Suprathel^®^) may also be conveyed to oral tissue regeneration. The animal experiment that we conducted comprised both an analysis of mucosal defect healing (after vestibuloplasty) and bone healing (postextraction alveoli healing).

After investigating the capacity of Suprathel^®^ to regenerate oral tissue – mucosa and bone, by juxtaposing it in a split-mouth animal model to Mucoderm^®^, a certified biomaterial for keratinized oral mucosa healing, no significant differences between these two biomaterials could be found in respect of all but one (profound epithelialization) of the investigated characteristics of guided tissue healing.

### Principles of guided tissue regeneration

There are numerous factors that influence the success of a regeneration procedure. They include factors linked to the properties of the regeneration membrane (barrier), such as barrier occlusion and stability, the size of the barrier perforations, peripheral sealing, adequate blood supply, access to tissue-forming cells (epithelium, bone and others) [24–27].

Resorbable membranes belong to the groups of natural or synthetic polymers. Collagen and aliphatic polyesters, such as polyglycolide or polylactide, are the most frequently used ones [28]. Collagen can be obtained from different sources and can be treated in various ways for membrane production. Polyglycolide or polylactide can be easily produced in different forms with multivalent physical, chemical and mechanical characteristics [29].

### Alloplastic materials

The most frequently used resorbable synthetic polymers in tissue engineering are poly-lactic acid (PLA), poly-glycolic acid (PGA), poly-lactide-co-glycolide (PLGA), polyvinyl alcohol (PVA), and polycaprolactone (PCL). There are several designs that stimulate uniform cell distribution, vascular supply, and cell differentiation used for tissue engineering: meshes, fibers, sponges, films, scaffolds and foams [30]. Among the multiple advantages of using these alloplastic materials, one can consider good biocompatibility, chemical versatility, favorable mechanical properties, low immunogenic reactions, and no risk of disease transmission [31]. They are degraded and eliminated through metabolic pathways [32]. The challenge is to design a structure that promotes cell differentiation, determining normal-tissue anatomy and function [33].

### Xenogeneic collagen matrix (Mucoderm®)

Guided tissue regeneration can also be consistently and predictably sustained with the help of collagen barriers of animal origin [34]. Angiogenesis is enhanced by collagen, which promotes healing and enhances fibroblast expression. It is resorbable, and there is no need for a second surgery [35]. The xenogeneic collagen matrix has in its composition collagen type I and III. It is a frequently used augmentation biomaterial to enhance soft peri-implant soft tissue, and it is used as a replacement for autogenous grafts [20, 36, 37].

Mucoderm^®^ (Xenogeneic collagen membrane) has a proven clinical use for increasing the keratinized mucosa width, including around dental implants, although it does not increase its thickness like the connective tissue graft [38].

### Suprathel®

The gold standard for regeneration of the deep dermal and full-thickness skin defects (burns, ulcers) is still considered the autologous spit-thickness skin graft [39, 40]. Nevertheless, several issues have been reported with the current use of skin grafts, and due to this, a variety of wound dressings have been developed [41]. Synthetic polymers, which the body degrades using enzymatic processes, have been introduced in many medical areas in recent years [42, 43].

Suprathel^®^ is a synthetic terpolymer mainly based on DL-lactide with trimethylene carbonate and ε-caprolactone. The final product is an interconnected porous membrane clinically successfully certified for skin healing. It shows large plasticity and adapts to the wound surface. It is water permeable, and it favors reepithelialization of wounds [44]. All of the components of Suprathel^®^ have valuable properties as regeneration biomaterials.

Lactic acid, lactate (anion), and lactide (di-ester derived from lactic acid) are known for their favorable properties in promoting tissue healing in general and skin formation in particular. The responsible mechanisms are believed to be multiple. It elicits angiogenesis via stimulation of growth factors, migration of endothelial cells, induces homing of stem cells, triggers diverse activation loops, and even modulates gene expression in human mesenchymal stem cells [45]. It accelerates the healing and has an antiseptic effect, whereas lactate-mediated oxidants stimulate fibroblasts, thus contributing to the formation of the dermis [46].

Various combinations of Suprathel^®^’s components have gained attention (e.g., polylactide-co-ε-caprolactone – PLLC blends with collagen) and were subjected to research in the physiologically relevant environment in which they would be utilized, particularly as natural biopolymers, sensitive to water content. Their properties might vary with hydration – e.g., blend elasticity increases with hydration [47, 48].

Although displaying longer healing times when compared to a skin graft, Suprathel^®^ demonstrated comparable early scar formation [49]. This material can serve as treatment for patients with extensive burns to cover the deep dermal burn wounds, saving donor sites for split-thickness skin grafts to be used in full-thickness burned areas [50]. Also, this membrane significantly reduces pain, is easy to handle, no allergic reactions are reported, and the material is easy and pain-free to remove after complete healing of the burns [50].

### Mucosal healing – epithelialization

Overall, our study showed no significantly different outcomes for the healing of mucosal defects covered with Suprathel^®^ and Mucoderm^®^, except for profound epithelialization, which occurred more often on the side where tissue regeneration was guided by Mucoderm^®^. Both materials favored the formation of a normal epithelium to similar degrees, whereas inflammation, simple granuloma, as well as foreign body granuloma, were present in comparable rates.

Several studies have compared tissue healing after free gingival graft vestibuloplasty, which is considered the gold standard, to porcine collagen matrix (Mucoderm^®^) with comparable results regarding the clinical and histological outcome. The main advantages of using collagen matrix to augment keratinized tissues surrounding dental implants are that they do not require a harvesting procedure, surgery time is reduced, and the regenerated tissues appear more esthetic. Tissue shrinkage has also been cited for both methods, with a higher degree in the collagen matrix group [51–53].

### Socket preservation – bone formation

After an extraction, the alveolar bone remodels, and the resorption process begins. Socket preservation diminishes this effect, and it usually involves placing biomaterials into the socket to support the remodeling process and coverage with a protective membrane in order to preserve bone tissues and minimize bone loss after teeth extraction [54]. Preservation has been known to produce less resorption when compared to normal healing after tooth extraction [55].

Guided bone regeneration (GBR) is a procedure based on the protected space theory in which a barrier membrane is used for space maintenance over a bone defect or the tooth alveolus specifically after extraction, promoting the ingrowth of osteogenic cells and preventing migration of undesired cells from the overlying soft tissues into the alveolus [56]. Resorbable and non-resorbable membranes have been used with good results in reducing bone resorption [57]. For non-resorbable membranes, a frequent complication is membrane exposure, with subsequently reduced rates of new bone formation [58]. Thus, resorbable membranes offer a viable alternative for this problem.

The need for the delayed placement of dental implants is an indication of socket preservation. It is generally performed with bone autografts, allografts (frozen-dried bone allograft, FDBA), xenografts (collagenated porcine bone, CPB), or alloplasts (hydroxyapatite, magnesium-enriched hydroxyapatite, calcium sulfate). FDBA [59] and CPB [60] have been shown to have a positive effect on height and width when compared to natural healing. The xenograft provides stability to the site due to its density and low resorption rate [61]. Another promising new approach is using concentrated growth factors (recombinant human bone morphogenetic protein – rhBMP-2) that can preserve the alveolar bone with almost no height change from baseline to 4 months [62].

An alternative to socket preservation therapy is implant placement with simultaneous contour augmentation using GBR. This technique described by Buser *et al.* involves extraction without flap elevation, debridement, followed by healing of the soft tissues for 4–8 weeks, implant placement with simultaneous contour augmentation on the buccal aspect with GBR using resorbable membranes combined with autogenous chips and a low resorption bone filler, as well as tension-free primary wound closure [63].

The advantages and disadvantages of the two major types of membranes used in guided bone regeneration procedures, including socket preservation, are shown in Table 3 (non-resorbable vs. resorbable membranes).

**Table 3.** Non-resorbable vs. resorbable membranes for alveolus bone regeneration.

Type of membrane	Advantages	Disadvantages	Commercial products
Non-resorbable	• May include the use of titanium; • Maintains shape until removal; • Can be easily fixed with titanium or bone tacks; • Regenerates more bone if complication-free; • No inflammatory response unless the membrane is exposed; • Numerous studies available.	• Need of an additional intervention for removal; • Increased patient morbidity; • Exposure leads to the necessity of removal; • Experience is required for optimal handling.	• ePTFE membranes; • Titanium reinforced membranes.
Resorbable	• No need of removal (cost effective); • Low morbidity; • Good soft tissue healing; • Membrane exposure does not mandate immediate removal; • Numerous studies demonstrate its validity.	• Variable resorption rate; • Uncertain barrier to specific tissues; • Difficult to handle; • Less bone regenerated than with non-resorbable membranes; • Resorption with various inflammatory response which may interfere with healing and GBR.	• Bovine collagen matrix ; • Porcine collagen matrix ; • Cross-linked collagen barrier;

Suprathel^®^ was investigated in this study concerning its capacity to act as a resorbable membrane for GBR. The present study found comparable bone healing characteristics in those alveoli that regenerated under Suprathel^®^ membranes compared to their corresponding split-mouth pairs for which healing occurred under Mucoderm^®^ membranes.

Both these biomaterials exhibited significantly improved healing and GBR characteristics regarding their socket-filling ability and their capability to foster the regeneration of normal bone trabeculae, with higher degrees of bone maturity, compared to the naturally healing control alveoli.

### Future developments

Research on tissue regeneration in the oral cavity is confronted with the multifaceted challenges of the complex oral environment – humidity, microbial flora, constant mechanical impact – conditions which render it highly demanding. As the processing of materials also plays an essential role in modulating their properties, extensive development of complex blends of polymers with natural tissue components and healing-promoters (collagen, fibrin, growth factors) in a hydrated environment has been undertaken in the attempt to obtain materials with improved and quantifiable physical and mechanical properties.

Electrospinning has recently gained attention as a source of an easy, cost-effective method for producing materials for medical purposes in a homogenous solution [47]. The electrospun scaffolds made for tissue engineering applications can be seeded with cells to recondition or replace tissue. By adding drugs into the electrospinning solution, drug delivery systems, such as implants, transdermal patches, oral forms, can be prepared. These scaffolds fulfill a similar purpose as the extracellular matrix in natural tissue. Polycaprolactone, a resorbable polymer, component of Suprathel^®^, is typically used for this purpose. The resulting compounds may then be coated with collagen to promote cell attachment, as collagen has already been spun into membranes with good results [64].

## Conclusion

This animal study achieved the validation of Suprathel^®^ (a resorbable material, already successfully used for skin regeneration) as a versatile and effective oral epithelium and bone regeneration material. The results of this study pave the way for future clinical trials that could further investigate the use in humans of this highly promising material for guided oral tissue and bone regeneration.

## Acknowledgments

### Ethical approval

The approval for this study was obtained from the Ethics Committee of the Iuliu Hatieganu University of Medicine and Pharmacy, Cluj-Napoca, Romania (approval no. 211/May 4^th^, 2018).

### Conflict of interest

The authors declare that there is no conflict of interest.
